# Integrated Analysis of Immune Infiltration Features for Cervical Carcinoma and Their Associated Immunotherapeutic Responses

**DOI:** 10.3389/fcell.2021.573497

**Published:** 2021-04-09

**Authors:** Yanan Kang, Jin Huang, Yang Liu, Nan Zhang, Quan Cheng, Yi Zhang

**Affiliations:** ^1^Department of Gynecology, Xiangya Hospital Central South University, Changsha, China; ^2^Department of Epidemiology and Health Statistics, School of Public Health, Central South University, Changsha, China; ^3^Hunan Provincial Key Laboratory of Clinical Epidemiology, Changsha, China; ^4^One-Third Lab, College of Bioinformatics Science and Technology, Harbin Medical University, Harbin, China; ^5^Department of Neurosurgery, Xiangya Hospital, Central South University, Changsha, China; ^6^Department of Clinical Pharmacology, Xiangya Hospital, Central South University, Changsha, China; ^7^National Clinical Research Center for Geriatric Disorders, Changsha, China

**Keywords:** cervical cancer, immunocytes, tumor microenvironment, immunotherapy, PD-1

## Abstract

Cervical cancer is the fourth most prevalent cancer in women, which decreases quality of life of the patients. Traditional interventions have failed to improve the overall survival period of patients due to high tumor recurrence after treatment or late diagnosis. Fortunately, preliminary evidence suggests that anti-angiogenic and immunotherapy can efficiently treat against cervical cancer. However, there is no clear evidence on the efficacy of immunotherapy in cervical cancer. Therefore, in this study, we classified cervical cancers in the TCGA dataset using various algorithms and explored the relationship between the immune profile and corresponding sensitivity of the tumors to immunotherapy. Results showed that patients with tumors had higher expression of immunocytes and longer overall survival time. In addition, we build a scoring system based on the immune landscape of the tumor microenvironment of cervical cancer. Tumors with higher scores exhibited better survival outcomes and were more sensitive to immunotherapy. In this study, the immune landscape of cervical cancer was analyzed, and the subtype of cervical cancer based on that difference was proposed. Besides, the subtype of cervical cancer showed different sensitivity to immunotherapeutic response which further confirmed its relationship with tumor immune landscape.

## Introduction

Cervical cancer is the fourth most prevalent cancer among women. In the developing countries, it is the leading cause of cancer-associated mortalities (Canfell, 2019). The median age for patients diagnosed with cervical cancer is 49 years. In general, cervical cancer lowers the quality of life of the affected persons (Canfell, 2019). It has been established that prolonged infection with human papillomavirus (HPV) type 16 and 18 is a risk factor for cervical cancer (Crosbie et al., 2013). Prophylactic vaccines against high-risk HPV types minimize the risk of developing cervical cancer. However, due to the limitations associated with HPV vaccines, reliable therapeutic options for cervical cancer, particularly recurrent or advanced tumors, are required (Shanmugasundaram and You, 2017). Current therapeutic options include surgical removal of the tumors based on the FIGO staging system, incorporated with chemo- or radiotherapies (Bhatla et al., 2019; Koh et al., 2019). As for recurrent cervical cancer, bevacizumab in combination with other therapies can significantly prolong patients’ survival time (Tewari et al., 2017). In addition, immunotherapy is a viable option for cervical cancer treatment.

Immunotherapy is effective against various solid tumors. Mechanistically, immunotherapy enhances immune responses by utilizing immune checkpoint inhibitors and adoptive cellular transfer (O’Donnell et al., 2019). However, immunotherapeutic depends on the tumor microenvironment (Gasser et al., 2017). Immunocyte infiltration degree, tumor mutational load, and T cell functions affect tumor sensitivity to immunotherapy. Programmed death ligand 1 (PD-L1) has been reported in over 90% of cervical cancer and tumors. Higher infiltration ratios of CD8+ T cells and CD4+ T regulatory cells confer better survival outcomes for tumor patients (Ramanathan et al., 2018; Otter et al., 2019). The immune system is also involved in HPV-induced tumorigenesis (Orbegoso et al., 2018). HPV has been known to trigger chronic inflammation, escape immune surveillance by hiding in keratinocytes, suppress cellar immunity, and wall itself with recruited immunocytes (Piersma, 2011). Based on these features, immunotherapy presents the best strategy for managing cervical cancer (Piersma, 2011; Smola, 2017; Kagabu et al., 2019).

Establishment of reliable biomarkers for the best choice of immunotherapy as well as an improved understanding of immune infiltration features with regard to cervical cancer are key to immunotherapy. Therefore, this study aimed at exploring cervical cancer-induced immune infiltration characteristics based on different clustering algorithms, with the aim of providing a strong foundation for research and rationale for immunotherapy. The ratio of immunocytes, overall survival outcomes, mutation burden, and immunotherapeutic responses between different groups were compared.

## Materials and Methods

### CESC Data and Preprocessing

A total of 291 samples from publicly available cell carcinoma (CESC) gene-expression datasets in The Cancer Genome Atlas (TCGA) were utilized in our analyses (Supplementary Table 10). The TCGA dataset was downloaded from UCSC Xena^1^, and subsequent analysis was performed using the R software (version 3.6.1) and R Bioconductor packages.

### Estimation of TME Infiltrating Cells

The respective proportions of the immune infiltrating cells in the cervical squamous CESC samples were quantified using the ssgsea algorithm (Hanzelmann et al., 2013). The gene sets comprised of 782 genes that could predict the abundance of 28 TIICs in individual tissue samples^2^. CESC was selected because it allows for the determination of sensitivity and specificity of immune cell phenotypes. It can be used to discriminate up to 28 human infiltration immune cell phenotypes.

### Unsupervised Consensus Clustering of TME-Infiltrating Cells

In order to generate more groups for further analyses, Partitioning Around Medoid (PAM) (Tahiri et al., 2018) was used to classify tumors with qualitatively diverse TME-infiltrating patterns. The optimal number of clusters in the TCGA cohort was determined using the ConsensuClusterPlus R package (Monti et al., 2003). The consensus ESTIMATE algorithm was performed to assess the infiltration of stromal and immune cells in CESC samples (Yoshihara et al., 2013).

### Identification of TME-Associated Differentially Expressed Genes

The patients were grouped into two distinct TME clusters based on the expression of immune infiltrating cells. The R package limma was used to determine differentially expressed genes (DEGs) between the two TME cell-infiltrating clusters (Ritchie et al., 2015). Adjusted *p*-value < 0.01 and | logFC| > 1 were considered to be statistically significant for DEGs between the TME subtypes.

### TME Gene Signatures Generations and Dimension Reduction

The DEGs between TME clusters were standardized for all the samples in the TCGA CESC cohort. Prognostic associated DEGs were filtered out by performing the Univariate Cox regression analysis (*p*-value < 0.05). The unsupervised clustering method (Hartigan and Wong, 1979) was used to classify patients into either of the TME gene clusters. Annotation of the TME gene pattern was performed using the clusterProfiler R package (Ghasemi and Zahediasl, 2012). The clustering algorithm (Monti et al., 2003) was used to define the gene clusters. Principal component one that served as the signature score was obtained using the principal component analysis (PCA). The TME score for each patient was determined based on the prognostic value of the gene signature (Sotiriou et al., 2006):

TME⁢score=∑PC1i-∑SPC1j

where “i” is the signature score of clusters with HR > 1, while “j” represents the expression of genes with HR < 1.

### Pathway Enrichment Analysis

The gene sets for pathway enrichment analysis were downloaded from the MSigDB database (Subramanian et al., 2005). Gene set variation analysis (GSVA) was performed on the TME score and the TME clusters using the clusterProfiler R package (Ghasemi and Zahediasl, 2012). Genes for the enriched Pathways in TME were identified using Gene Ontology (GO) and Kyoto Encyclopedia of Genes and Genomes (KEGG), with an adjusted *p* < 0.05.

### Immunotherapeutic Response Prediction

The Tumor Immune Dysfunction and Exclusion (TIDE) algorithm was used to link individual responses to immunotherapeutic responses (Jiang et al., 2018; Lu et al., 2019). Differences in anti-PD-1 and CTAL-4 therapeutic response were evaluated using the Submap analysis. For the melanoma data set (GSE78220, *N* = 28), the expression profiles (FPKM normalized) of GSE78220 were transformed into TPM values which were then used to calculate the TME score (Wagner et al., 2012). With regard to the urothelial cancer data set (IMvigor, *N* = 298), the data package was downloaded from http://research-pub.gene.com/IMvigor210CoreBiologies. Quality control and trimming of the mean of M-values were performed using the R package arrayQualityMetrics to normalize the numerical data (Ritchie et al., 2015).

### Statistical Analysis

The Shapiro–Wilk normality test was used to establish variable normality (Ghasemi and Zahediasl, 2012). For normally distributed variables, the unpaired Student *t*-test was used to compare differences between the two groups, whereas the Wilcoxon test was used to compare abnormally distributed variables. One-way analysis of variance (ANOVA) and Kruskal–Wallis tests were used for comparison of multiple groups.

Pearson and distance correlation analyses were performed to calculate correlation coefficients. The χ^2^ contingency test was performed to determine the interrelationships between TME score and anti-PD-1 response. The overall survival and TME score were determined using the R package. The threshold for survival values was determined. Based on the dichotomized TME score, patients were grouped into either high or low TME clusters, while at the same time reducing the computational batch effect by the R package sva. The data were visualized using the ggplot2 for R package. In the analysis of differential gene expression, we used the Benjamini–Hochberg method that converts *p*-values to FDRs to identify significantly expressed genes (Schreiber et al., 2011). OncoPrint was used to delineate the mutation landscape in the TCGA dataset using the maftools R package (Wang et al., 2020). Survival curves for the subgroups were generated using the Kaplan–Meier method. Statistical significance between different data sets was determined using the log-rank test. The univariate and multivariate Cox proportional hazard regression models were performed using the R package to determine independent factors associated with prognosis. Survivorship curves were generated using the R package survminer. Heatmaps were generated based on pheatmap. All statistical analyses were performed using R^3^. The tests were two-sided, with *p*-values < 0.05 being considered to be statistically significant.

## Results

### The Landscape and Functional Annotation of CESC TME

**FIGURE 1:**
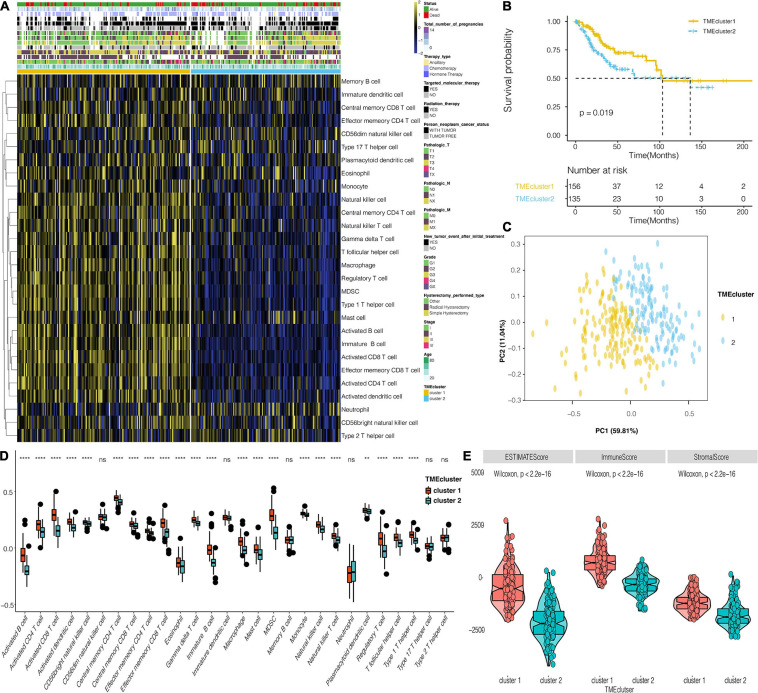
The landscape of CESC TME, and the characteristics of TME subtypes in TCGA. **(A)** Unsupervised clustering of TME cells for 291 patients in TCGA. **(B)** Kaplan–Meier curves for 291 patients in TCGA, showing the association between TME infiltration patterns and OS (log-rank test, *P* < 0.001). **(C)** PCA for the two TME clusters. **(D)** The distribution of immunocytes in the two TME clusters. Within each group, the scattered dots represent values for cellular expression at TME whereas the thick line represents the median value. The bottom and top (lines) in the boxes are the 25th and 75th percentiles (interquartile range). The whiskers encompass 1.5 times the interquartile range. The statistical difference of the two TME clusters was compared using the Kruskal–Wallis test. **, *P* < 0.01; ****, *P* < 0.0001; ns, not statistically significant. **(E)** The differential expression of Estimate, Immune, and Stromal Score for TME clusters in the TCGA dataset.

**FIGURE 2:**
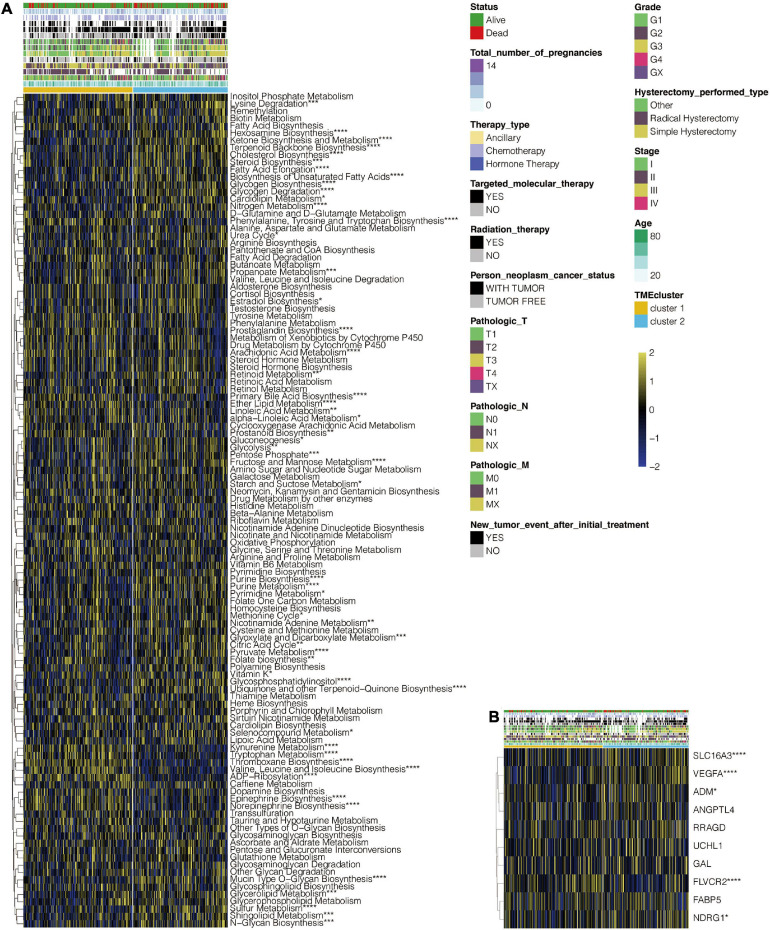
Functional annotation of TME clusters. **(A)** Heatmap depicting the correlation of TME clusters and metabolic pathways. **(B)** Heatmap depicting the correlation of TME clusters and hypoxia-related gene signatures. *, *P* < 0.05; **, *P* < 0.01; ***, *P* < 0.001; ****, *P* < 0.0001; ns, not statistically significant.

The flowchart for this study is shown in Supplementary Figure 1A. Analysis on cluster stability performed on CESC in the TCGA dataset using ConsensusClusterPlus package to select the optimal cluster number is shown in Supplementary Figure 1B. PAM of the 291 tumors with corresponding TME cell expression profiles in the TCGA cohort on its part is shown in Figure 1A. Two TME phenotypes were established based on immune cell infiltration. They conferred significantly different OS for outcomes (log-rank test, *p* < 0.001) as shown in Figure 1B. PCA showed a clear separation between the two established groups in the TCGA dataset (Figure 1C). Figure 1D shows the distinct TME infiltration patterns for the two clusters. Based on the ESTIMATE algorithm, TME cluster 1 was strongly associated with the estimated, immune, and stromal scores compared with TME cluster 2 (Figure 1E). Furthermore, 20 immune-related signaling pathways and DNA regulation-related pathways in GO analysis (Supplementary Table 1) were identified in the TCGA data set (Supplementary Figure 2A). Intrinsic immune escape was attributed to the expression of seven immune checkpoint molecules including antigen presenters, co-stimulators, co-inhibitors, receptors, ligands, and cell adhesion proteins, among others (Schreiber et al., 2011; Wang et al., 2020). There was an elevated expression of immune checkpoint molecules around TME cluster 1 of the CESC that aid the respective tumor cell escape from immune killing in TCGA (Supplementary Figure 2B). Moreover, the correlation of TME clusters, hypoxia, and metabolism was explored. TME cluster 2 was found to correlate with more metabolism-related signaling pathways (Figure 2A). TME cluster 2 also positively correlated with hypoxia-related gene signatures, indicating a malignancy of TME cluster 2 (Figure 2B).

### Generation of TME Gene Signatures and Functional Annotation

**FIGURE 3:**
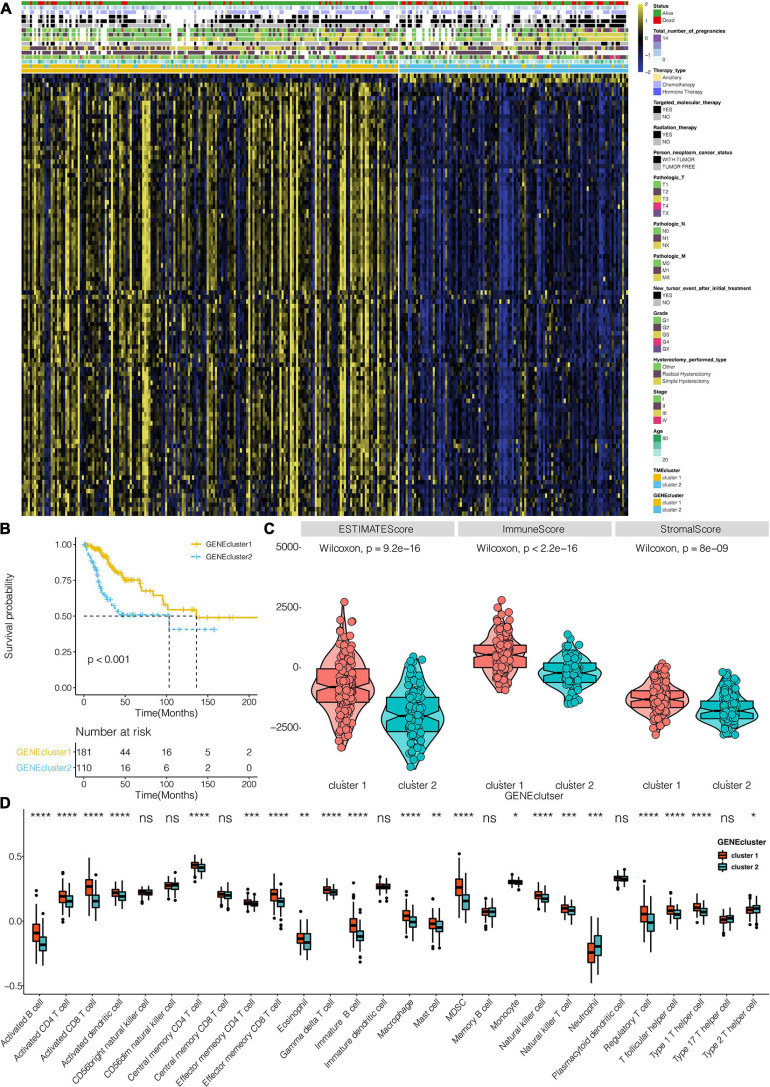
TME signatures and functional annotation constructs. **(A)** Unsupervised analysis and hierarchical clustering of common DEGs based on expression data of CESC derived from the TCGA: Gene clusters 1 and 2. **(B)** Kaplan–Meier curves for the two TME gene clusters (log-rank test showed an overall *P* < 0.001). **(C)** The differential expression of Estimate, Immune, and Stromal Score in TME clusters in the TCGA dataset. **(D)** The distribution of cells in TME gene clusters. *, *P* < 0.05; **, *P* < 0.01; ***, *P* < 0.001; ****, *P* < 0.0001; ns, not statistically significant.

**FIGURE 4:**
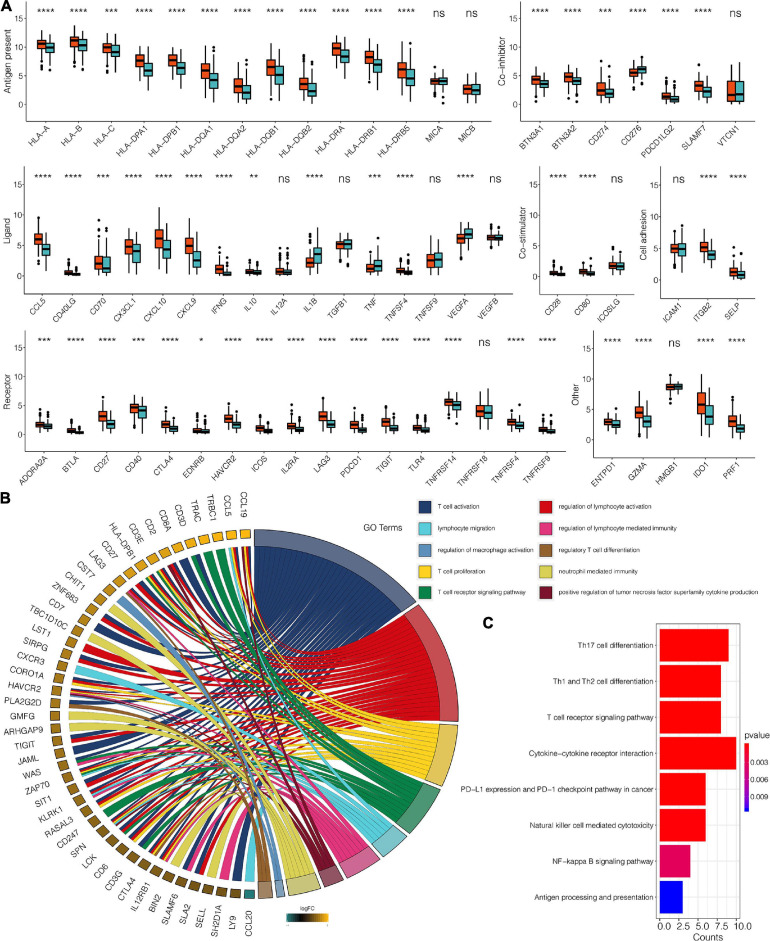
**(A)** The expression pattern of seven types of immune checkpoints in TME gene clusters for the TCGA dataset. **(B)** The GO enrichment analysis of the 98 DEG for TME signatures. **(C)** KEGG enrichment analysis of the 98 DEG for TME signatures. *, *P* < 0.05; **, *P* < 0.01; ***, *P* < 0.001; ****, *P* < 0.0001; ns, not statistically significant.

A total of 383 DEGs (Supplementary Table 2) were identified and used to classify the patients into genomic types, to further investigate the potential biological characteristics of each TME infiltration cell pattern. The clustering stability established by ConsensusClusterPlus package for the optimal number of clusters (Supplementary Figure 1C) was in tandem with the two CESC gene clusters (gene clusters 1 and 2) generated in TCGA (Figure 3A). Survival analysis of the two clusters revealed that the expression of gene cluster 1 was associated with better survival outcome (Figure 3B). Compared to cluster 2 genes, cluster 1 gene expressions were also associated with immune, stromal, and estimate scores (Figure 3C). Furthermore, cluster 1 genes were correlated with higher expression levels of infiltrating immune cells (Figure 3D). Compared to cluster 2 genes, CESC in gene cluster 1 were significantly associated with the immunosuppressive process that was mediated by a higher expression of immune checkpoint molecules (Figure 4A).

**FIGURE 5:**
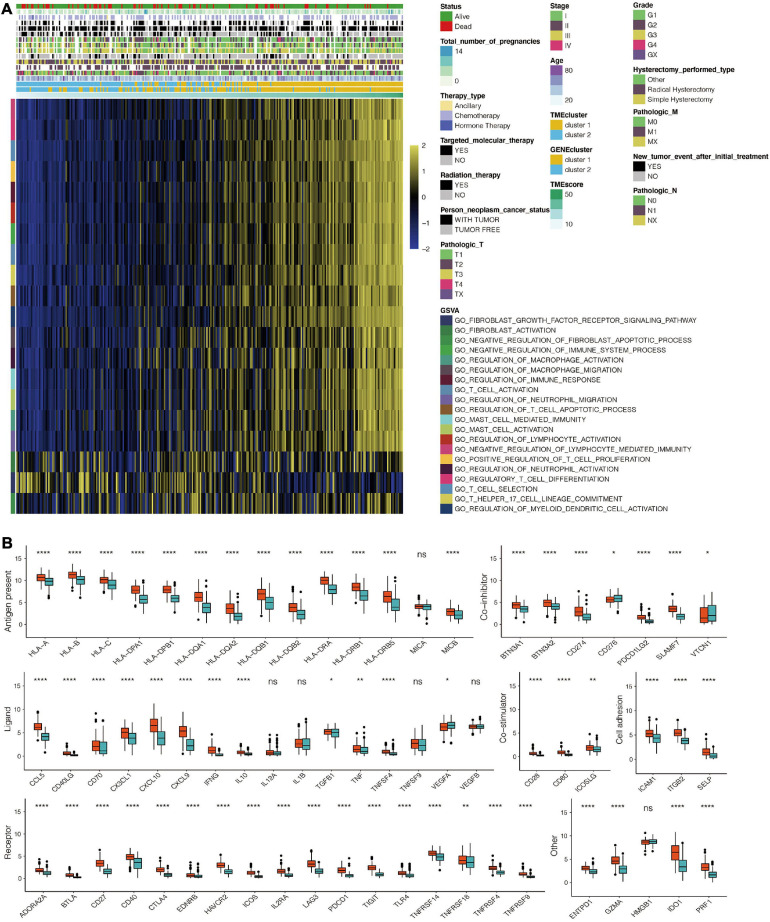
Immune-related functional annotation for the TME score. **(A)** GSVA for the TME score based on GO for TCGA. **(B)** The expression pattern of seven types of immune checkpoints in the TME score-based clusters for TCGA. *, *P* < 0.05; **, *P* < 0.01; ***, *P* < 0.001; ****, *P* < 0.0001; ns, not statistically significant.

**FIGURE 6:**
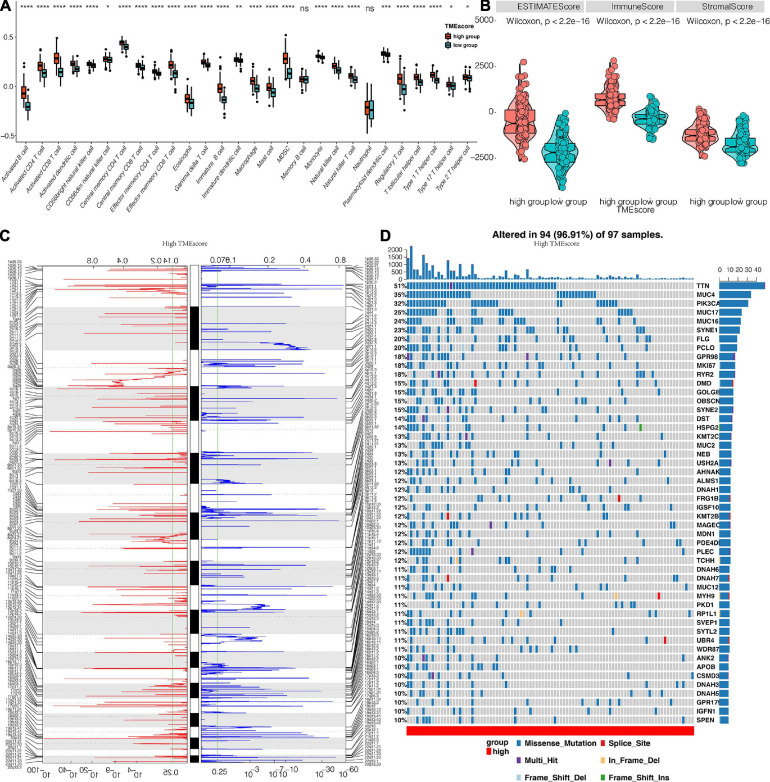
Genomic profiles associated with the TME score. **(A)** The distribution of immune cells in TME score clusters for TCGA. **(B)** The differential expression of Estimate, Immune, and Stromal Score for TME score in the TCGA dataset. **(C)** GISTIC 2.0 amplifications and deletions in CESC with high TME score. Chromosomal locations of peaks of significantly recurring focal amplification (red) and deletions (blue). **(D)** Differential somatic mutations in CESC with a high TME score. *, *P* < 0.05; **, *P* < 0.01; ***, *P* < 0.001; ****, *P* < 0.0001; ns, not statistically significant.

Univariate Cox regression analysis for TME scores and the corresponding transcriptome traits as well as clinical characteristics for the top98 DEGs are shown in Supplementary Table 3, respectively. GO enrichment analysis revealed that 98 genes were associated with T cell activation and proliferation, regulation of macrophage activation, positive regulation of tumor necrosis factors, and neutrophil-mediated immunity. Combined, these pathways regulate the immune system (Figure 4B and Supplementary Table 4). KEGG enrichment analysis was consistent with GO analysis, with 98 genes found to be associated with immune system regulation (Figure 4C and Supplementary Table 5). TME scores for patients with CESC in the TCGA dataset are shown in Supplementary Table 6. GO analysis revealed that high TME scores were significantly associated with immune-related pathways, including T cell selection, T cell activation, regulation of macrophage activation, negative regulation of lymphocyte-mediated immunity, mast cell activation, regulation of myeloid dendritic cell activation, regulatory T cell differentiation, and immune response activation (Figure 5A and Supplementary Table 7). Furthermore, CESC with high TME scores exhibited higher immune checkpoint expression levels (Figure 5B). High TME scores were also associated with macrophage, mast cells, MDSC, and regulatory T cell infiltration, all of which induce an immunosuppressive environment. Moreover, a high TME score was also a predictor for a more activated immune environment. This is because high TME scores were correlated with a higher infiltration of multiple T cells and natural killer cells (Figure 6A). The two-sided role of high TME score could be attributed to the complexity of multiple immune cell-infiltrated tumor microenvironment. High TME scores were also positively correlated with Estimate, Immune, and Stromal Score (Figure 6B).

### TME Score Is Associated With Unique Patterns of Genomic Alterations

CNA and somatic mutation analysis performed on the TCGA dataset to determine the association between TME score and CESC genomic profiles revealed that samples with high TME scores frequently amplified several genomic regions particularly drivers of oncogenesis and immune regulatory genes including NRAS (1p13.27), DUP3Q29 (3q29), LYZ (5p11), HLA-DQA1 (6p21.32), CHEK2P2 (15q11.1), STAT3 (17q21.2), and KLK3 (19q13.33). These gene sets were associated with COL11A1 (1p21.1), MCL1 (1q21.2), UGT2B7 (4q13.2), ANGPT2 (8p23.1), PTEN (10q23.31) TNFRSF13B (17p11.2), TNNI3 (19q13.42), and GSTT1 (22q11.23) gene deletions as shown in Figure 6C. Different genomic profiles were observed in low TME score as shown in Supplementary Figure 3A. Furthermore, somatic mutation profiling revealed a high frequency of mutations in TTN (51%), MUC4 (35%), PIK3CA (32%), MUC17 (25%), MUC16 (24%), and SYNE1 (23%) among genes with high TME score (Figure 6D), whereas TTN (32%), MUC4 (29%), PIK3CA (29%), and MUC16 (27%) were the frequently mutated genes in the low TME score cluster (Supplementary Figure 3B).

### TME Score Predicts Therapeutic Responses

**FIGURE 7:**
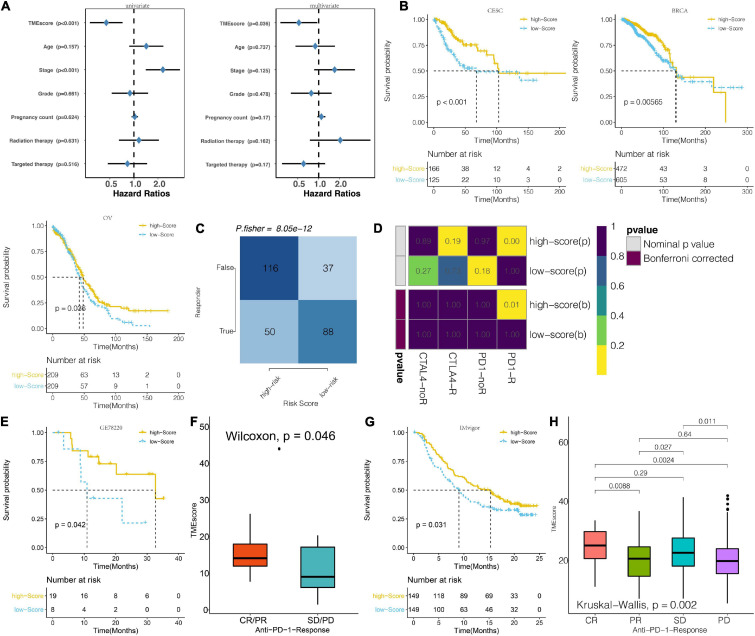
Prognosis potential of TME score for immunotherapeutic response. **(A)** Univariate and multivariate Cox regression model estimating prognostic potential of TME score, patient age, tumor stage, tumor grade, pregnancy count, radiation therapy, and targeted therapy in TCGA. (The length of horizontal line represents the 95% confidence interval for each group. The vertical dotted line represents the HR of all patients. HR < 1.0 indicates that a high TME score is a biomarker for positive prognosis). **(B)** Kaplan–Meier curves for the two groups of patients classified along the TME score for CESC, BRCA, and OV from the TCGA. (Log-rank test showed an overall *P* < 0.001). **(C)** The TIDE value and response to immunotherapy for the TME score clusters. **(D)** Submap analysis based on the TIDE algorithm for differential response to CTAL-4 and anti-PD-1 therapy with regard to the TME score for the TCGA dataset. **(E)** Kaplan–Meier curves for the two groups of melanoma patients classified based on TME score for GSE78220. (Log-rank test showed an overall *P* < 0.001). **(F)** TME scores for groups with different anti–PD-1 clinical response status (CR/PR and SD/PD) in the GSE78220 dataset. (Wilcoxon, *P* = 0.019). **(G)** Kaplan–Meier curves for the two groups of patients classified based on the TME score in the IMvigor cohort. (Log-rank test: *P* < 0.001). **(H)** Distribution of TME scores in groups with different anti–PD-L1 clinical response statuses in the IMvigor cohort.

In the analysis of the association between prognosis and TME score for the CESC cohort, it was found that high TME scores and targeted therapy were respective markers and intervention for positive prognosis of CESC in the TCGA dataset. Univariate and multivariate analyses revealed that patient ages, tumor stage, and radiation therapy were correlated with poor CESE prognosis (Figure 7A). A high TME score was associated with better survival outcomes for patients with CESC, BRCA, and OV in the TCGA dataset (Figure 7B). TME scores for BRCA and OV patients in the TCGA dataset included in this study are shown in Supplementary Tables 8, 9, respectively. Analysis of the probable response to immunotherapeutic response for CESE in the TCGA based on the TIDE algorithm revealed that low TME score tumors responded better to immunotherapy compared to high TME score tumors (Figure 7C). Subsequently, anti-PD-1 and anti-CTLA-4 therapeutic responses were analyzed. Tumors in the two TME score clusters were found to respond differently to immunotherapies. Tumors in the high TME score clusters exhibited a better response to anti- than those with low TME scores (Figure 7D). In the melanoma dataset, GSE78220 patients with high TME scores exhibited significantly longer OS outcomes than patients with lower TME scores (Figure 7E). High TME scores were also correlated with complete and partial anti-PD-1 responses (Figure 7F). In the urothelial cancer dataset, patients with high TME scores exhibited significantly longer OS outcomes than those with low TME scores in the IMvigor210 cohort (Figure 7G). Additionally, high TME scores were also associated with strong anti-PD-1 responses (Figure 7H).

## Discussion

The component of the cervical cancer microenvironment can affect cervical cancer progression, but potential mechanisms are still elusive. In this study, we analyzed the influence of immunocyte infiltration shed on tumor response to immunotherapy by applying different algorithms and proposed the subtype of cervical cancer based on immunocyte infiltration. CESC in the TCGA dataset were grouped into two clusters based on the differential expression of immunocytes, and lower immunocyte infiltration ratio samples showed worse survival outcome. In the meantime, several metabolic related pathways and hypoxia-associated genes were also differentially activated or expressed between the low-risk group (TME cluster 1) and the high-risk group (TME cluster 2). Therefore, the immune subtype of cervical cancer, TME cluster 1, and TME cluster 2 can affect the formation of the tumor microenvironment and cervical cancer progression.

To step further, 383 DEGs in total were identified and used to deeply explore this immune subtype of cervical cancer. High TME score group samples are usually accompanied with higher infiltration ratios of immunocytes like NK cells, T cells, and macrophages and higher immunotherapy-related gene expression (like HLA-A, HLA-B, HLA-C, CCL5, CXCL10, CD40, CTLA4, and PDCD1). In addition, immune activation-related pathways are also differentially activated in the low and high TME score groups. The previous study reported that chemokines like CCL5 and CXCL10 can modulate tumor sensitive to immunotherapy (Vilgelm and Richmond, 2019). Biomarkers like CTLA4, CD40, and the HLA family have been confirmed to be involved in immune surveillance (Waldman et al., 2020). Therefore, tumors with a low TME score group may be more sensitive to immunotherapy than high-scoring samples. However, its prediction ability still requires being examined with more clinical samples.

Previous studies also discussed the association between cervical cancer and its immune landscape. A previous study analyzed the proportion of immunocytes in cervical cancer and identified prognostic related immunocytes (Wang et al., 2019). Another study stratified samples based on the expression profile of differentially expressed immune-related genes and suggested that samples with higher infiltrated CD8 T cells and mast cells are more sensitive to immune checkpoint inhibitors (Yang et al., 2019). Moreover, tumor mutation loads are also critical intrinsic factors that affect tumor response to immunotherapy (Gasser et al., 2017; Havel et al., 2019). For instance, the amplification of HLA-DQA1 and STAT3 (Bae et al., 2020; Zou et al., 2020) and the deletion of ANGPT2 and TNFRSF13B (Rotolo et al., 2016; Lauret Marie Joseph et al., 2020) from the high TME score group have been proved to be immunotherapy-associated factors. Therefore, the TME score may serve as a potential tool to evaluate the tumor sensitivity to immunotherapy.

Previous studies proposed the “hot” and “cold” tumor analogy to describe tumor sensitivity to immunotherapy (Galon et al., 2006; Galon and Bruni, 2019). Given that tumors with high TME scores exhibited higher infiltrations of activated immunocytes and inflammatory related cells, tumors in this group may be referred as “hot” tumors. Moreover, high TME score tumors exhibited better PD-L1 receptor therapeutic response than low TME score tumors. Thus, adopting different strategies may improve patients’ clinical outcome. For instance, T-cell-targeted therapy (Buchbinder and Desai, 2016; Hellmann et al., 2016) or microbiome modulation (Snyder et al., 2015; Routy et al., 2018) was recommended to “hot” tumors. Chemotherapy, in combination with T cell enhancement or stimulatory signals, can improve “cold” tumor sensitivity (Whiteside et al., 2016). Taking the TME score into consideration in the choice of cervical cancer treatment may improve patients’ survival outcome.

In this study, infiltration of activated immunocytes was preferentially enhanced in the high TME score group. A higher immunocyte infiltration and enhanced immune checkpoint gene expression in the high TME score cluster implies that these tumors are more sensitive to immunotherapy. In conclusion, this study highlights the impact of the tumor microenvironment to immunotherapeutic sensitivity in tumors.

## Data Availability Statement

The datasets presented in this study can be found in online repositories. The names of the repository/repositories and accession number(s) can be found in the article/Supplementary Material.

## Author Contributions

YK: data collection, analysis, and manuscript preparation. JH, YL, and NZ: manuscript revision. QC and YZ: study design. All authors contributed to the article and approved the submitted version.

## Conflict of Interest

The authors declare that the research was conducted in the absence of any commercial or financial relationships that could be construed as a potential conflict of interest.

## Footnotes

1 https://xenabrowser.net/

2 http://software.broadinstitute.org/gsea/msigdb/index.jsp

3 https://www.r-project.org/, version 3.6.1

## Supplementary Material

Supplementary Figure 1 Flow chart and cluster analysis related information. **(A)** The diagrammatical flow chart for this study. **(B)** TME clusters; TCGA matrixes for each k (*k* = 2–4), displaying the clustering stability using 1,000 iterations of hierarchical clustering. **(C)** TME gene clusters; TCGA matrixes for each k (*k* = 2–4), displaying the clustering stability using 1000 iterations of hierarchical clustering.

Supplementary Figure 2 GSVA analysis and immune landscape based on TME clusters. **(A)** GSVA of TME clusters in TCGA-GO **(B)**. The expression pattern of seven types of immune checkpoints in TME clusters for TCGA.

Supplementary Figure 3 Genomic profiles associated with TME score. **(A)** GISTIC 2.0 amplifications and deletions in CESC with high TME score. Chromosomal locations of peaks of significantly recurring focal amplification (red) and deletions (blue). **(B)** Differential somatic mutations in CESC with high TME score.

Supplementary Table 1 GO pathways for TME clusters based on GSVA in TCGA.

Supplementary Table 2 383 differentially expressed genes in TCGA.

Supplementary Table 3 98 representative TME signature genes.

Supplementary Table 4 GO enrichment analysis for TME gene clusters based on 98 genes.

Supplementary Table 5 KEGG enrichment analysis for TME gene clusters based on 98 genes.

Supplementary Table 6 TME score for CESC patients in TCGA.

Supplementary Table 7 GSVA of TME score.

Supplementary Table 8 TME score for BRCA patients.

Supplementary Table 9 TME score for OV patients.

Supplementary Table 10 Characteristics of all the included patient samples.
