# Supplementary material for: Integrated Analysis of Immune Infiltration Features for Cervical Carcinoma and Their Associated Immunotherapeutic Responses
Source: Front Cell Dev Biol. 2021 Apr 9;9:573497. doi: 10.3389/fcell.2021.573497 (PMC8063060; doi:10.3389/fcell.2021.573497)
Supplement: Supplementary Figure 1 — Flow chart and cluster analysis related information. (A) The diagrammatical flow chart for this study. (B) TME clusters; TCGA matrixes for each k (k = 2–4), displaying the clustering stability using 1,000 iterations of hierarchical clustering. (C) TME gene clusters; TCGA matrixes for each k (k = 2–4), displaying the clustering stability using 1000 iterations of hierarchical clustering. [file Data_Sheet_1.ZIP › supplementary figure.docx]

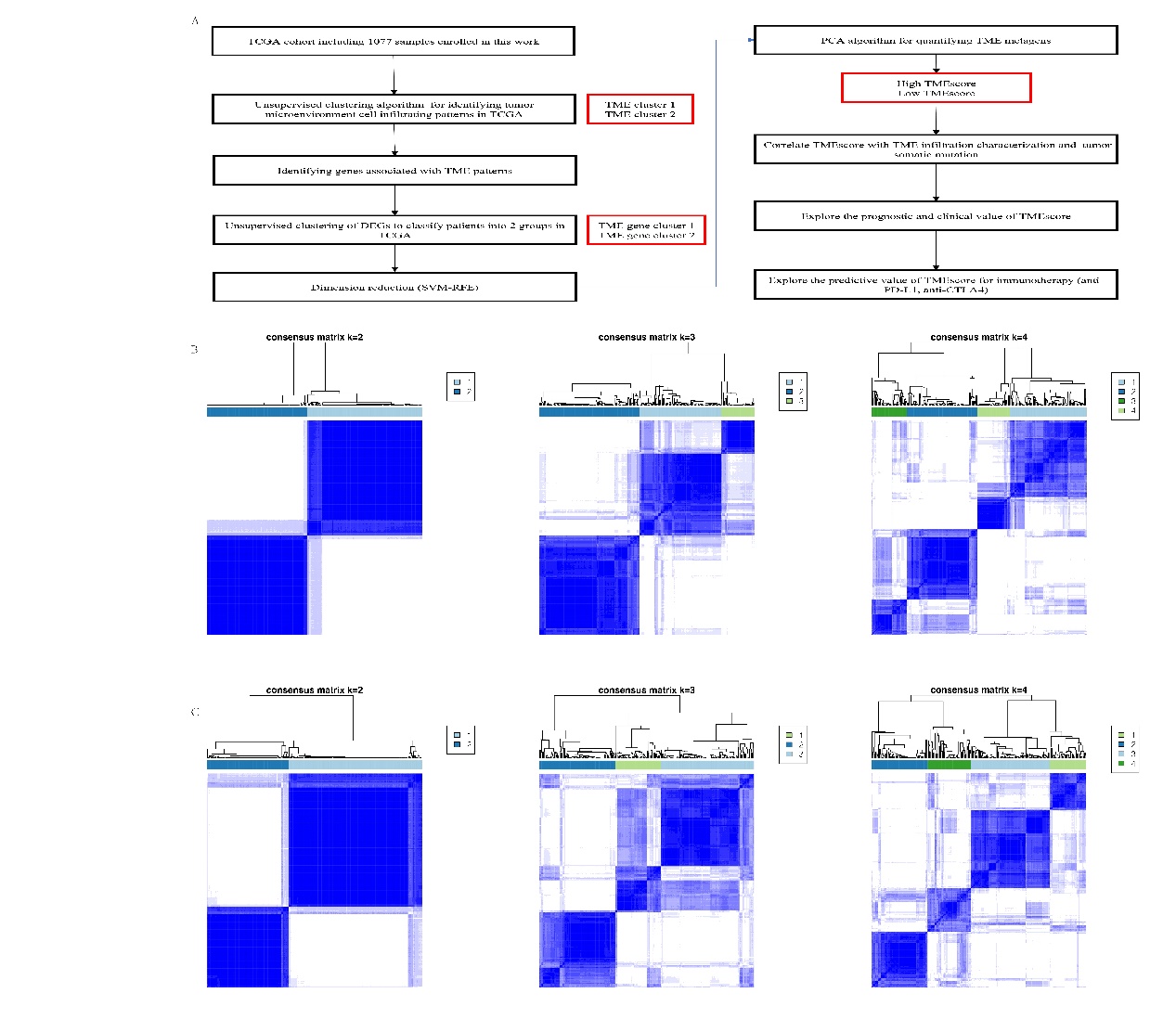


Figure S1: A. The diagrammatical flow chart for this study. B. TME clusters; TCGA matrixes for each k (k = 2–4), displaying the clustering stability using 1000 iterations of hierarchical clustering. C. TME gene clusters; TCGA matrixes for each k (k = 2–4), displaying the clustering stability using 1000 iterations of hierarchical clustering.


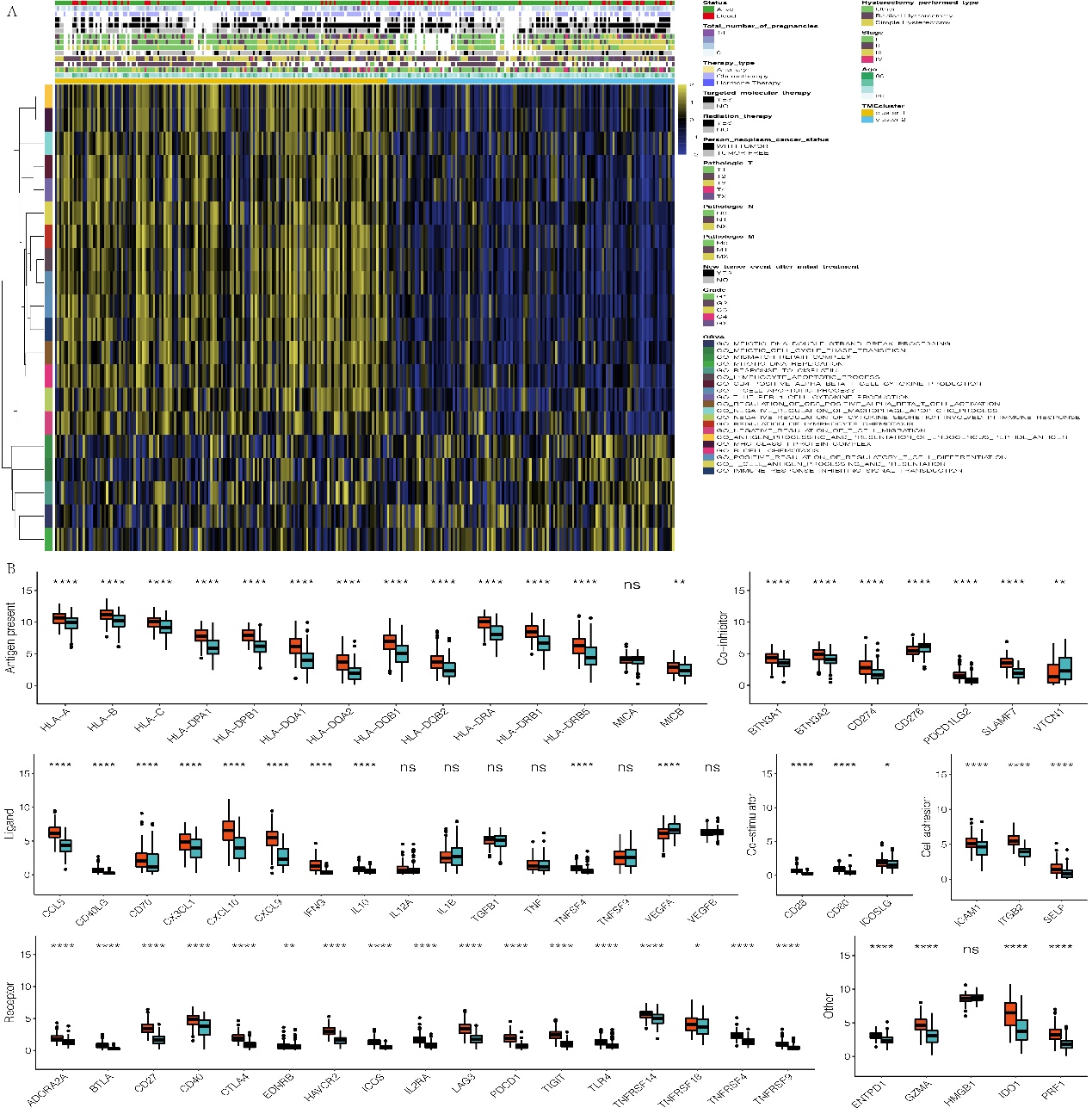


Figure S2: A. GSVA of TME clusters in TCGA-GO B. The expression pattern of seven types of immune checkpoints in TME clusters for TCGA.


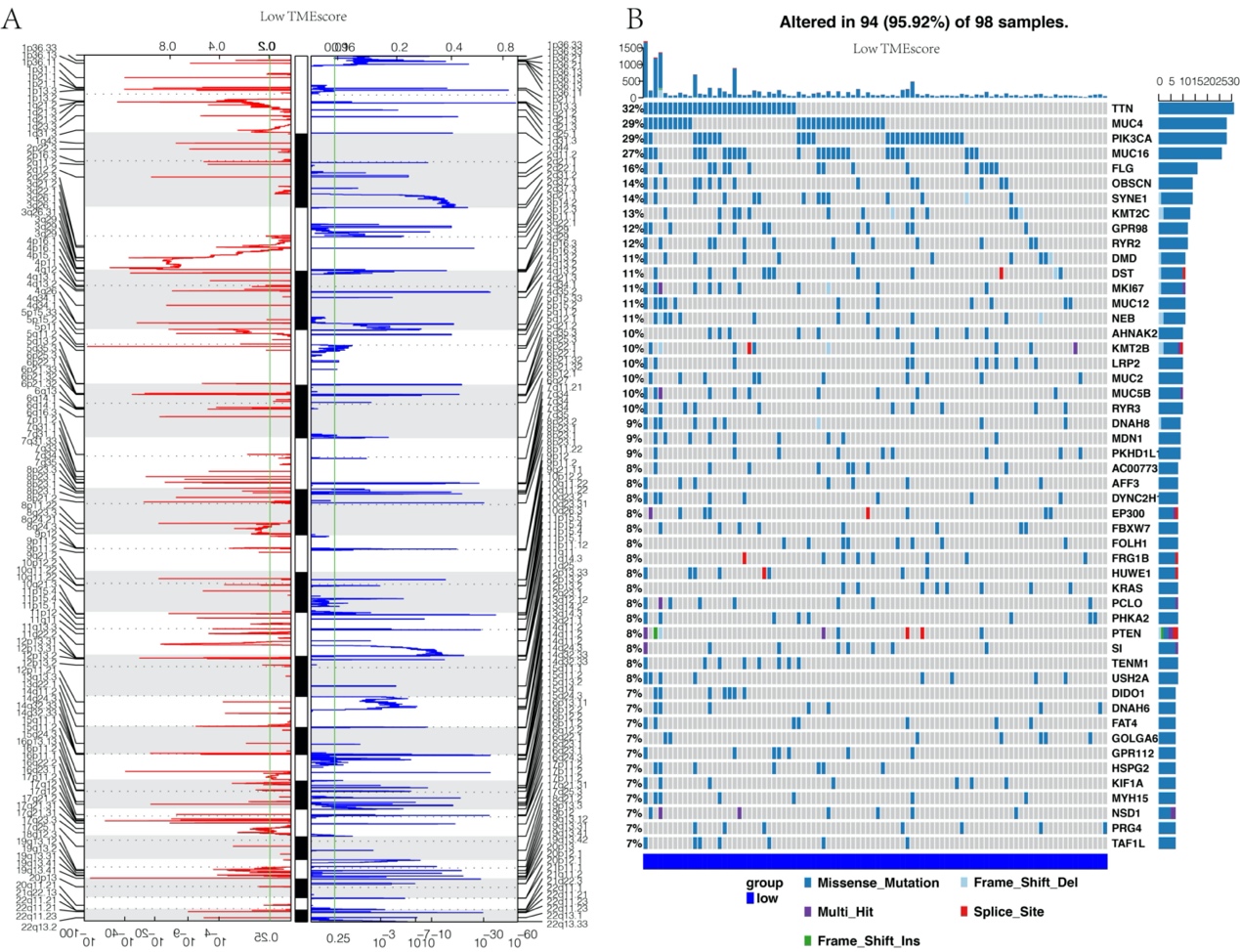


Figure S3: Genomic profiles associated with TME score. A. GISTIC 2.0 amplifications and deletions in CESC with high TME score. Chromosomal locations of peaks of significantly recurring focal amplification (red) and deletions (blue). B. Differential somatic mutations in CESC with high TME score.
